# Scapular dislocation following radical surgical excision of lung sarcomatoid carcinoma: A rare case report

**DOI:** 10.1097/MD.0000000000036884

**Published:** 2024-01-12

**Authors:** Qiang Wu, Kun Qiao, Jixian Liu, Shuo Zhen, Zizi Zhou

**Affiliations:** a Department of Cardio-Thoracic Surgery, Shenzhen University General Hospital, Shenzhen, China; b Department of Thoracic Surgery, National Clinical Research Center for Infectious Disease, Shenzhen Third People’s Hospital, Shenzhen, China; c The Second Affiliated Hospital, School of Medicine, Southern University of Science and Technology, Shenzhen, China; d Department of Thoracic Surgery, Peking University Shenzhen Hospital, Shenzhen, China.

**Keywords:** case report, lung sarcomatoid carcinoma, radical surgical excision, scapular dislocation

## Abstract

**Rationale:**

Scapular prolapse is a rare complication of thoracotomy. Only a few cases of scapular prolapse after thoracotomy have been reported. Here, we report the case of a 52-year-old male patient who underwent standard posterior thoracotomy for lung sarcomatoid carcinoma invading the left upper chest wall.

**Patient concerns:**

The surgery was performed to remove some ribs and chest wall muscles; however, no reconstruction or repair of the chest wall defect was performed. The patient experienced a sharp pain and severe limitation of movement of the left shoulder within 1 month of receiving adjuvant therapy.

**Diagnoses:**

The patient was diagnosed with left intrathoracic scapular prolapse after careful consideration of medical history, physical examination, and chest radiography.

**Interventions:**

We performed closed manual reduction because the patient refused to undergo surgery.

**Outcomes:**

The patient’s shoulder pain and movement limitation were significantly relieved, but the symptoms relapsed. After repeated closed manual reduction, the patient was instructed not to abduct the shoulder joint above 90°. The patient did not relapse during a 1-year observation period.

**Conclusion:**

If scapular prolapse occurs, manual or surgical reduction can be selected based on the needs. If a patient refuses to undergo surgery, manual reduction can be an effective treatment method.

## 1. Introduction

Scapular prolapse, also termed locked scapula or scapulothoracic dislocation, is a rare complication of thoracotomy.^[1]^ It can be divided into intrathoracic scapular prolapse and extrathoracic scapular prolapse, based on the relative positions of the scapula and thoracic cavity.^[2,3]^ Only a few cases of scapular prolapse after thoracotomy have been reported. The occurrence of this complication is relatively accidental and easy to miss with nonspecific symptoms of shoulder pain and movement limitation, which makes it difficult to diagnose, and a more detailed medical history, physical examination, and radiological examination are needed.^[4,5]^ Here, we describe a patient with lung sarcomatoid carcinoma who had scapular prolapse after thoracotomy, and discuss the diagnostic and therapeutic processes.

## 2. Case presentation

**Figure 1.**
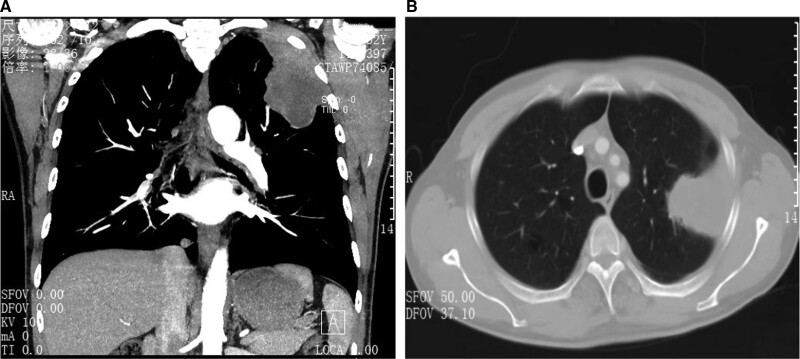
Chest computed tomography demonstrating the tumor in the left upper lobe of the lung invading the chest wall and 3^rd^ to 5^th^ ribs. (A) Coronal section (B) Transverse section.

A 52-year-old male visited our hospital due to left-sided chest pain for approximately 4 months. He had a history of smoking for 30 years. Except for this, he had no other medical history of note, and no abnormalities were found on physical examination. Chest computed tomography (CT) showed a subpleural mass with a diameter of 8 cm in the left upper lobe, which may have invaded the chest wall (Fig. 1A and B). The patient was examined using emission computed tomography of the bone, and no metastatic carcinoma of the rib was found. The patient underwent an open left upper lobectomy. During the operation, the mass was found to have invaded the left upper chest wall involving the 3rd, 4th, and 5th ribs. Therefore, the left 3^rd^ to 5^th^ ribs and the chest wall muscles were completely removed. No reconstruction and repair of the chest wall was performed during the operation, considering that the chest wall defect was small and could be covered by the scapula. Histopathological examination showed a sarcomatoid carcinoma of the left lung with tumor node metastases of pT4N1M0 stage IIIA.

**Figure 2.**
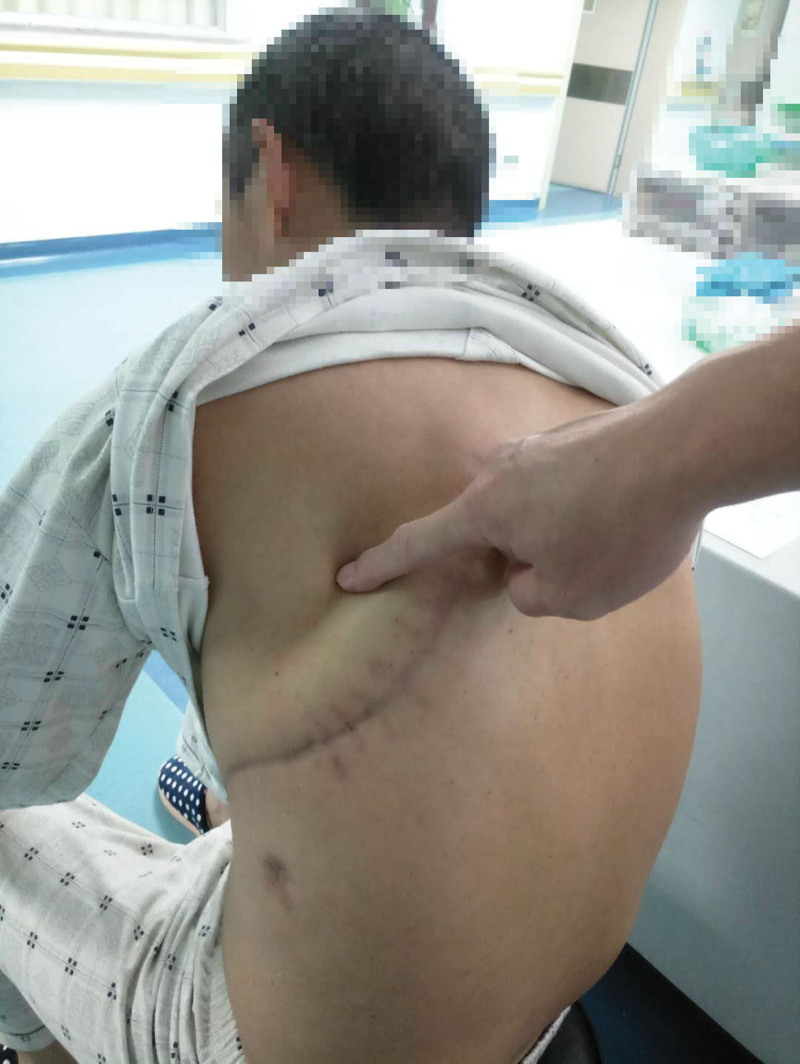
The left side of the patient’s scapular region is visibly depressed.

**Figure 3.**
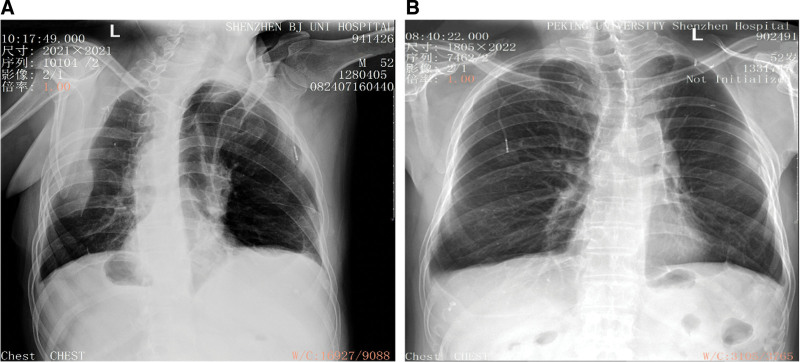
(A) Collapse of the upper left chest suggests a dislocation of the left scapula into the left pleural cavity. (B) After closed reduction of the scapular prolapse, we can see that the left scapula has been restorated on the chest radiography.

The patient recovered well postoperatively and received concurrent chemoradiotherapy. The patient experienced sharp pain in the left posterior chest wall with limited movement of the left upper limb within 1 month of receiving adjuvant therapy and was initially diagnosed with shoulder joint dislocation. Physical examination revealed left-sided scapular depression (Fig. 2), and chest radiography revealed a homogeneous shadow with a clear border in the left chest, indicating intrathoracic scapular displacement (Fig. 3A). The patient was diagnosed with left intrathoracic scapular prolapse. After closed manual reduction, symptoms such as shoulder pain were significantly relieved, and shoulder mobility improved (Fig. 3B). Scapular prolapse recurred after 1 month, and the patient underwent a repeat closed manual reduction. We noticed that scapular prolapse was prone to relapse when the shoulder was abducted > 90°. The patient was instructed to avoid excessive left shoulder abduction and had no scapular prolapse during a 1-year follow-up.

## 3. Discussions

Scapular prolapse is a rare complication of thoracotomy with complex causes, which are still not clearly understood, and few cases have been reported.^[6,7]^ Previous studies showed that patients with chest wall defects are prone to have scapular prolapse,^[8]^ but shoulder deformities, shoulder weakness, abnormal movement, chemoradiotherapy, steroids, and muscle relaxant are also considered as possible causes of scapular prolapse.^[9–11]^ We suspected that the enlarged intercostal space caused by the removal of some ribs was the main cause of scapular prolapse for this patient, and the removal of the serratus anterior, trapezius, rhomboid space, latissimus dorsi, and other chest wall muscles during the operation was also one of the causes of the prolapse since the loss of the chest wall muscles may affect the location, stability, and movement of the scapula. In addition, steroids were used as an adjuvant therapy, which may be another cause.

However, the exact cause of scapular prolapse after thoracotomy remains unclear. Early identification of scapular prolapse is important in patients with nonspecific shoulder pain, deformity, weakness, and movement limitation after thoracotomy. Chest radiography is one of the clues that can display soft tissue density surrounding the scapula and the location and angle of the scapula. Scapular prolapse is obvious on chest CT, especially after 3D reconstruction, as the inferior angle of scapular dislocation into the thoracic can be clearly displayed.^[12]^ Chest CT can also detect possible complications, such as rib fractures, pulmonary contusions, pneumothoraces, and hemothoraces.^[13]^

Unfortunately, a chest CT was not considered in the case of our patient when the scapular prolapse occurred. Regarding the treatment of scapular prolapse, Gould et al^[14]^ reported that closed manual reduction was performed for patients with poor general health instead of surgical reduction; however, failure of closed manual reduction still impaired the patient’s shoulder. Palissery et al^[15]^ reported 1 case of scapular prolapse selected for surgical reduction and reconstruction of the chest wall defect after repeated closed manual reduction. Akinori performed surgical reduction for patients with repeated scapular prolapse and proposed that the preventive operation should be performed during the primary operation if the chest wall defect could result in the scapular prolapse.^[16]^ Thanissara reported a 76-year-old female patient with scapular prolapse after lung transplantation who received closed manual reduction, restriction of arm movement for 2 weeks, and strengthening of the periscapular muscles, and the patient had no pain or shoulder movement limitation during follow-up.

Although closed manual reduction was effective, he believed that surgery for repair of the chest wall defect is required for unstable scapular prolapse.^[17]^ Takashi Eguchi reported another case of scapular prolapse that underwent surgical reduction due to scapular prolapse with active bleeding in the thoracic cavity, and the symptoms of shoulder pain and its movement limitation disappeared after the operation. Therefore, he believed closed manual reduction is limited in the treatment of scapular prolapse, and surgical reduction should become the priority choice if the patient can tolerate the operation.^[18]^ This opinion is not unique. R. S. Lee also reported that only manual reduction could not replace the scapula in a safe and stable location since the chest wall defect cannot maintain the scapula in most cases. Therefore, he recommended that surgical reduction should be the first-line treatment.^[19]^

However, Masaki reported a 64-year-old male patient with scapular prolapse after thoracotomy who experienced scapular prolapse after the operation, even though reconstruction and repair of the chest wall defect were performed during the operation.^[20]^ Youval believed that the conservative method is more popular for single scapular prolapse, while for patients with complications or repeated scapular prolapse, the operation is a better choice, undoubtedly.^[21]^ In our case, several ribs were removed since the tumor had invaded the chest wall, and no reconstruction and repair of the chest wall defect was performed. The combined effects of these factors resulted in scapular prolapse. We performed a closed manual reduction considering the patient’s locally advanced tumor, limited life expectancy, weakness caused by postoperative adjuvant treatment, and unwillingness to undergo surgery for reconstruction and repair of the chest wall defect. After reduction, shoulder pain was relieved, and shoulder movement was no longer limited; however, the symptoms relapsed easily. The patient was instructed not to abduct the shoulder joint > 90° after reduction. The patient followed our recommendations and showed no scapular prolapse during a 1-year follow-up period. Herein, we discuss the diagnosis and treatment of scapular prolapse after thoracotomy.

## 4. Conclusion

In patients experiencing nonspecific shoulder pain and movement limitations following thoracotomy, scapular prolapse should be strongly suspected. A thorough evaluation, including detailed medical history, positive signs, and chest radiological examination, is essential for accurate diagnosis. To mitigate the risk of scapular prolapse, intraoperative reconstruction and repair of chest wall defects should be prioritized during thoracotomy procedures. In cases where scapular prolapse occurs, the choice between manual or surgical reduction should be based on individual needs and preferences. This study emphasizes that manual reduction can be an effective treatment option, particularly when surgical intervention is declined by the patient. By implementing these strategies, healthcare professionals can improve the management and outcomes of scapular prolapse in thoracotomy patients.

## Author contributions

**Conceptualization:** Qiang Wu, Kun Qiao, Zizi Zhou.

**Data curation:** Qiang Wu, Kun Qiao, Zizi Zhou.

**Formal analysis:** Qiang Wu, Kun Qiao, Zizi Zhou.

**Funding acquisition:** Zizi Zhou.

**Investigation:** Qiang Wu, Zizi Zhou.

**Methodology:** Qiang Wu, Kun Qiao, Zizi Zhou.

**Project administration:** Qiang Wu, Kun Qiao.

**Resources:** Qiang Wu, Kun Qiao, Zizi Zhou.

**Software:** Qiang Wu.

**Writing – review & editing:** Jixian Liu, Shuo Zhen, Zizi Zhou.
